# Tilt aftereffect following adaptation to translational Glass patterns

**DOI:** 10.1038/srep23567

**Published:** 2016-03-23

**Authors:** Andrea Pavan, Johanna Hocketstaller, Adriano Contillo, Mark W. Greenlee

**Affiliations:** 1 University of Lincoln, School of Psychology, Brayford Pool, Lincoln LN6 7TS, United Kingdom; 2 University of Regensburg, Institute for Experimental Psychology, Experimental and Clinical Neuroscience Study Programme, Universitätsstr. 31, 93053 Regensburg, Germany; 3 University of Ferrara, Department of Physics and Earth Sciences, Via Saragat 1, 44122 Ferrara, Italy

## Abstract

Glass patterns (GPs) consist of randomly distributed dot pairs (dipoles) whose orientations are determined by specific geometric transforms. We assessed whether adaptation to stationary oriented translational GPs suppresses the activity of orientation selective detectors producing a tilt aftereffect (TAE). The results showed that adaptation to GPs produces a TAE similar to that reported in previous studies, though reduced in amplitude. This suggests the involvement of orientation selective mechanisms. We also measured the interocular transfer (IOT) of the GP-induced TAE and found an almost complete IOT, indicating the involvement of orientation selective and binocularly driven units. In additional experiments, we assessed the role of attention in TAE from GPs. The results showed that distraction during adaptation similarly modulates the TAE after adapting to both GPs and gratings. Moreover, in the case of GPs, distraction is likely to interfere with the adaptation process rather than with the spatial summation of local dipoles. We conclude that TAE from GPs possibly relies on visual processing levels in which the global orientation of GPs has been encoded by neurons that are mostly binocularly driven, orientation selective and whose adaptation-related neural activity is strongly modulated by attention.

The adaptation paradigm has been widely used to investigate the spatiotemporal properties of orientation selectivity mechanisms and their interactions. In a typical orientation adaptation paradigm, a prolonged exposure (adaptation) to an oriented visual stimulus causes a subsequent stimulus (test pattern) to appear rotated away from the adapting orientation. This phenomenon is known as tilt aftereffect (TAE)^1^,^2^,^3^,^4^,^5^,^6^,^7^,^8^,^9^. The direction and the magnitude of the orientation shift in the TAE depend on the relative orientation between the adapting and test stimuli^3^. Orientation differences between 0° and 50° lead observers to perceive the test pattern as oriented opposite to that of the adapting pattern. This is called the direct TAE or repulsion effect^10^ and peaks at an adapting-test orientation difference between 10° and 20° with a magnitude of ~4 deg^1^,^2^,^3^,^4^,^5^,^6^,^7^,^8^,^9^. On the other hand, if the relative orientation between adapter and test stimuli is larger than 50°, the test stimulus appears tilted towards the adapting orientation. This is known as the indirect TAE or attraction effect^11^. The peak of the attraction effect is usually at an adapting-test orientation difference of 75°–80° and on average has a magnitude of ~0.5 deg^12^. A computational model of the TAE outlined by Clifford *et al.*^2^ showed that both direct and indirect TAE can be explained by means of the overlap of two adapting effects, which they refer to as centering and scaling. The former inhibits the response of the neurons tuned to stimuli oriented in parallel to that of the adapting stimulus, causing a repulsion of the perceived test orientation. The latter broadens the bandwidth of the neurons tuned obliquely to the adapting stimulus, resulting in an attraction towards the oblique orientation. The overall result is a net repulsive effect in the region of small orientation differences, followed by a much weaker attractive one in the complementary region. Such scaling and centering constitute the model representation of the mechanisms of self-calibration and decorrelation particular to sensory cells.

Psychophysical research has shown that the direct TAE is reduced when the adaptation and test grating differ in spatial frequency^13^, showing spatial frequency selectivity and thus suggesting the involvement of early stages of visual processing. Indeed, there is physiological evidence that TAE depends on changes of orientation-selective cells in visual areas with orientation selective neurons. Fang *et al.*^14^ provided fMRI evidence in humans of orientation selectivity in visual areas V1, V2, V3/VP, V3A and V4, thus supporting the existence of adaptable, orientation-tuned neurons in the striate cortex and extrastriate visual areas.

To date TAEs have been reported using a variety of visual stimuli including oriented gratings^5^,^9^, single lines^1^,^8^,^15^, oriented stimuli defined by subjective contours^16^,^17^, texture edges defined by orientation contrast^16^, bilateral symmetrical patterns^18^ and orientation signals defined by fast moving dots (i.e., motion streaks^19^). Taken together these studies suggest that, depending on the adapting stimulus, the TAE may rely either on the striate cortex (V1) or on extrastriate areas (e.g., on V2 or higher extrastriate areas). Indeed, there is physiological evidence that neurons in the extrastriate cortex of macaque monkeys can be activated by subjective contours^20^,^21^,^22^. In the present study we measured the TAE from adaptation to stationary oriented translational Glass patterns (GPs). GPs^23^ contain randomly distributed dot pairs (dipoles), the orientation of which are determined by certain geometric transforms^24^,^25^,^26^,^27^,^28^. There is psychophysical evidence that translational GPs involve spatial summation across multiple local detectors responding to oriented dipoles^25^,^29^,^30^. The rationale of these studies was to investigate whether adapting to stimuli that require spatial integration across multiple local oriented filters in order to extract the global orientation, biases the response of orientation selective units. Smith and colleagues^31^,^32^ reported that macaque monkeys’ V1 and V2 neurons (simple and complex cells) show orientation selectivity for both gratings and translational GPs presented in their classic receptive field (CRF), although the response to GPs is generally weaker than the response to gratings. In addition, V1 and V2 neurons process similarly the sparse local orientation cues in translational GPs and both V1 and V2 neurons are not sensitive to global form information present in GPs stimuli extending outside the CRF. In general, V1 receptive fields are quite small compared with the GPs typically used in psychophysical experiments and so would typically encompass only a small part of the pattern. Moreover, the local cues for orientation in GPs are quite weak, because each dot pair is embedded in a random noise background. The absence of strong local contours means that the first stage of orientation-selective cells in the cortex might provide sparse, irregular signals and these signals need to be integrated by neurons tuned to global form^31^,^32^.

At least for circular GPs the detection is thought to be carried out over at least two stages. The first stage analyses the sparse local orientation information from dipoles and in a second stage local orientation cues are integrated in a global form percept^28^,^33^,^34^. However, as of date the processing stage at which translational oriented GPs are analyzed has not been clearly specified. A brain imaging study of Ostwald and colleagues^35^ found higher fMRI selectivity for translational GPs at lower stages of visual analysis (e.g., V1/V2), although pattern classification accuracy showed that translational GPs activates a wide range of cortical areas including V1, V2, V3, V3a, VP/V3, V4 and LOC. This could be compatible with the two-stage model proposed by Wilson *et al.*^28^ and Mandelli and Kiper^34^. On the other hand, there is more recent and contradictory brain imaging evidence in humans that early visual areas can process complex and global form. Mannion *et al.*^36^, using fMRI, found that for polar GPs, cortical areas V1, V2, V3 and hV4 show enhanced response for dipole orientations that are tangential to the fixation point. This enhanced response to tangential orientations indicates sensitivity to curvature and global form as early as striate cortex.

In order to further investigate the processing of translational GPs, we hypothesized that if adaptation to static oriented translational GPs produces an angular function similar to that described by previous TAE studies that used gratings or single lines as adapting stimuli^2^, this would indicate that translational GPs activate orientation selective mechanisms at a low level of visual processing, or at least at a level where neurons are still orientation selective (i.e., up to area V4, according to Fang *et al.*^14^). Any deviation from the angular function of the TAE would indicate that translational GPs are analysed at a level in which neurons are tuned for the global form and no longer selective for orientation. In the first experiment, we examined whether adaptation to stationary oriented translational GPs was able to bias the perceived orientation of a subsequently flashed grating. To anticipate our findings, the results show that, although reduced in magnitude, adaptation to translational GPs produced a significant direct TAE and a weak indirect TAE. These results are compatible with the involvement of detectors tuned for orientation. In a second experiment, we used the interocular transfer procedure (IOT) to assess whether adaptation to translational GPs rely on monocular or binocular units. The results showed an almost complete IOT for GP suggesting the involvement of orientation selective units that are mostly binocularly driven. In the third experiment, we investigated the effect of distraction on the magnitude of the TAE. There is physiological evidence that focal attention enhances the activity of V1, V2 and V4 neurons when a specific orientation is attended^37^. In addition, there is psychophysical evidence that focal attention to an oriented adapting grating increases the magnitude of the TAE^38^. Therefore, we investigated whether distraction during adaptation affects the TAE from both GP and grating adapters. The results showed similar distraction-related TAE reduction for GP and grating adapters. An additional experiment on GPs aimed to test whether distraction affects the spatial summation of local dipoles, indicated that the perceived global form of oriented translational GPs is not influenced by attentional modulation. Based on these results we speculate that translational GPs are analysed at a level in which (i) the global orientation is encoded, (ii) neurons are mostly binocularly driven but still orientation selective, and (iii) adaptation-specific neural activity is strongly modulated by attention.

## Experiment 1: Tilt aftereffect from translational stationary GPs

### Methods

#### Participants

One of the authors (JH) and seven naïve participants took part voluntarily in the experiment, and all received compensation for their time (except for the author). All participants had normal or corrected to normal visual acuity. Viewing was binocular. Methods were carried out in accordance with the Declaration of Helsinki (1964). This study was approved by the Ethics Committee of the University of Regensburg (http://ethikkommission.uni-regensburg.de/) (reference number: 13-101-0029). Written informed consent was obtained from each participant prior to the enrolment in the study.

#### Apparatus

Stimuli were displayed on a 23-inch Samsung T23A750 monitor with a refresh rate of 60 Hz. Stimuli were generated with Matlab PsychToolbox^39^,^40^. The screen resolution was 1920 × 1080 pixels. Each pixel subtended 1.6 arcmin at the used viewing distance (see below). The minimum and maximum luminance of the screen were 0.22 and 88.07 cd/m^2^ respectively, and the mean luminance was 42.8 cd/m^2^. Luminance was measured with a photometer (OP200-E, Cambridge Research System Ltd, Rochester, Kent, UK). A gamma-corrected lookup table (LUT) was used so that luminance was a linear function of the digital representation of the image luminance levels. Observers sat in a dark room at a distance of 57 cm from the screen. The participant’s head was stabilized by asking her/him to rest her/his chin on a chinrest. A black cardboard with a 20 cm circular aperture was placed on the computer screen to remove any vertical and horizontal cues. Stimuli were seen through the circular aperture.

#### Stimuli

**Figure 1:**
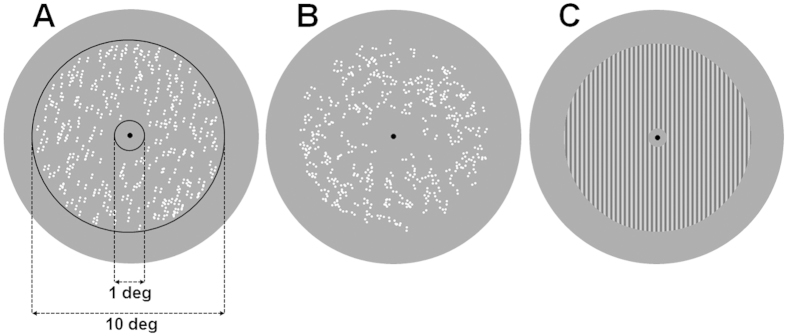
Stimuli used in Experiment 1. (**A**) An adapting translational GP oriented at 15° clockwise from vertical. The black circular frames, not presented during the experiment, delimit the circular annulus in which GPs were presented. (**B**) An adapting noise GP; dipoles have random orientation sampled from a uniform distribution. (**C**) A vertical test grating with a spatial frequency of 4.12 c/deg.

Adapting stimuli were oriented translational GPs made up by 300 pairs of dots (dipoles) with an inter-dot separation of 0.18 deg. The diameter of the dots was 0.12 deg. The dipoles were randomly placed in a circular annulus with the radius of the larger circle of 5 deg and the radius of the smaller inner circle of 0.5 deg. The dipole density of the GPs was 1.9 dipoles/deg^2^. All dipoles had the same orientation (i.e., 100% coherence). We adapted to seven oriented translational GPs: 0° (vertical), 15°, 30°, 45°, 60°, 75° and 90° (horizontal)^2^,^9^,^19^. Translational GPs were oriented clockwise from vertical. In order to estimate the subjective vertical, observers were also adapted to noise GPs in which each dipole had a random orientation from a uniform distribution between 0° and 360°, thus resulting in GPs with 0% orientation coherence (Fig. 1B). The test pattern was a circular annulus sinewave grating of the same size of the adapting pattern (i.e., radius of the outer and inner circles 5 and 0.5 deg, respectively) and with a spatial frequency of 4.12 c/deg. The spatial frequency of the grating matched the spatial extent of the dots in the adapting GPs (i.e., a bar of the grating was ~0.12 deg in width). The test grating had a fix contrast of 0.5 (Michelson contrast). Figure 1 shows an example of the adapting and test stimuli used in Experiment 1.

#### Procedure

In Experiment 1 we measured TAE from adaptation to oriented translational GPs. We used a top-up adaptation procedure in which an adapting translational GP was presented initially for 30 s and then for 10 s on subsequent trials. After the adapting period and an adaptation-test blank interval of 16 ms, in which only the central fixation point was presented, a test grating was flashed for 33 ms. A tone was produced 1 s before the onset of the test stimulus to prevent participants from blinking. We used a very brief test grating for two reasons: the first was that TAE is stronger for very brief test durations^41^ and secondly we assessed whether adaptation to oriented translational GPs biases the perceived orientation of an oriented stimulus with stronger local oriented contours^19^,^31^. Observers had to judge whether the test grating was tilted either clockwise or counter-clockwise from vertical (Method of Single Stimuli [MSS]^42^) by pressing one of two designated keys on a standard computer keyboard (the key “Left Arrow” was used to indicate counter-clockwise orientation, and the key “Right Arrow” for clockwise orientation). A simple up/down staircase^43^ was used to estimate for each observer the point of subjective verticality (PSV) of the test pattern. On the first trial of each staircase, the orientation of the test pattern was chosen randomly between −2.5° and 2.5° from vertical (negative values represent counter-clockwise orientations from vertical). On subsequent trials, the test grating was rotated in the direction opposite to that reported by the observer, such that the orientation of the test stimulus converged to the PSV, i.e., the orientation for which observers were at chance in discriminating whether the test pattern was oriented clockwise or counter-clockwise from vertical. The test grating was rotated by 2° until the first reversal in observers’ response, 1° until the second reversal, 0.5° until the third reversal, 0.25° until the fourth reversal, and 0.1° thereafter until 14 reversals had been made. The staircase terminated either after 100 trials or 14 reversals. The adapting orientation was kept constant within each staircase. The PSV was estimated by averaging the orientation values of the test grating corresponding to the last 10 reversals. For each adapting orientation observers performed two staircases. The PSV values estimated from the two staircases were averaged, and then we subtracted the subjective vertical estimated following adaptation to the noise GPs; this removed any bias in observers’ orientation judgement^16^,^18^,^19^. The resulting value was the magnitude of the TAE (in deg) for each adapting orientation. The procedure used in the control condition to estimate the subjective vertical (i.e., the baseline) was identical to that used to estimate PSV. However, for the baseline condition observers performed 3 to 4 staircases. The resulting PSV values were then averaged and the resulting value was considered as the subjective vertical.

One staircase lasted approximately five to six minutes. Wolfe and O’Connell^44^ found that TAE of adaptation to luminance contours drops off rapidly, returning to less than a quarter of its maximum after 5 min, with minimal long-term adaptation. Accordingly, observers were required to rest approximately 5 min between each staircase to control for cumulative adaptation effects. As an additional precaution against cumulative adaptation effects the sequence, in which adaptation to the oriented and the noise GPs was conducted, was randomized.

### Results

**Figure 2:**
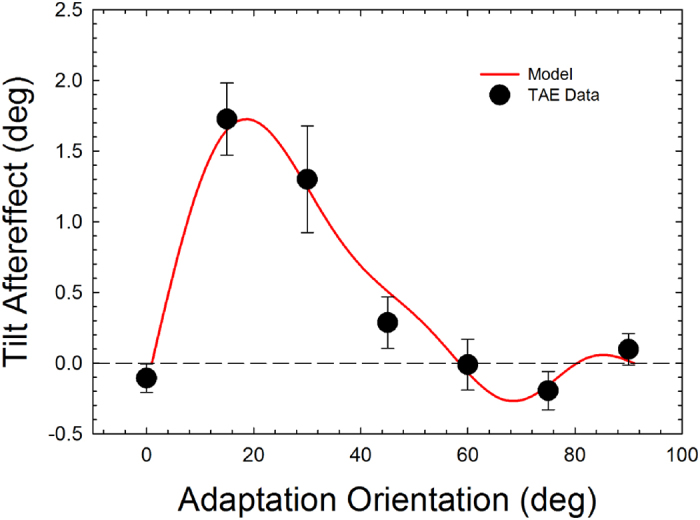
The tilt aftereffect (TAE) (in deg) is shown as a function of the adapting orientation (black circles). The peak repulsive effect (direct TAE) was obtained following adaptation at 15°. The results are shown for N = 8 participants. The red line refers to the TAE predicted by the best fitting model. Error bars ± SEM.

Figure 2 (black circles) shows the results of Experiment 1. A Kolomogorow-Smirnov test for normality revealed that data were not normally distributed (*p* < 0.001). A nonparametric Friedman test revealed a statistically significant difference in tilt bias depending on the adapter orientation (*χ*^2^_6_ = 27.64, *p* = 0.0001).

The resulting function is asymmetrical and “S” shaped, with the sign of the function changing beyond 60° of orientation adaptation. An additional analysis of variance for repeated measures with trend analysis revealed a significant linear (*F*_1,7_ = 22.77, *p* = 0.002, *partial-η*^*2*^ = 0.77), quadratic (*F*_1,7_ = 9.16, *p* = 0.019, *partial-η*^*2*^ = 0.57), cubic (*F*_1,7_ = 64.69, *p* = 0.0001, *partial-η*^*2*^ = 0.90) and quartic trends (*F*_1,7_ = 21.45, *p* = 0.002, *partial-η*^*2*^ = 0.75). Higher order trends were not significant (quintic, *F*_1,7_ = 1.61, *p* = 0.24, *partial-η*^*2*^ = 0.19; sextic, *F*_1,7_ = 0.36, *p* = 0.57, *partial-η*^*2*^ = 0.048). These results are compatible to those reported by van der Zwan and Wenderoth^9^. In their experiments observers were adapted to purely subjective contours.

In order to test whether adaptation to 15° and 75° oriented GPs produced significant tilt biases (i.e., repulsive and attractive, respectively) we performed Bonferroni corrected planned one-sample t-tests between the estimated TAEs and zero (*p*_*crit*_ = 0.025). The one-sample t-tests revealed a significant direct TAE (*t*_7_ = 6.78, *p* = 0.0001), but a non-significant indirect TAE (*t*_7_ = −1.44, *p* = 0.19).

### Discussion

The angular function observed by adapting to oriented translational GPs, although reduced in magnitude, is similar to those reported when adapting to oriented gratings or lines^2^,^3^,^7^,^45^,^46^, subjective contours^9^,^16^,^47^,^48^, contours defined by orientation contrast^16^ and adaptation to fast directional moving dots producing motion streaks^19^. In particular, we found significant repulsion effects (or direct TAE) but not a significant attraction effect (indirect TAE).

The near-identical pattern of tuning implies that oriented translational GPs are likely to be encoded by the same orientation-selective neurons present in early visual cortical areas^31^,^32^, thought to underlie the classic TAE^2^,^17^,^41^. To further assess whether TAE from translational GPs involves the same orientation selective mechanisms and inhibitory lateral interactions of TAE obtained from luminance contours, we fitted our data with the low-level computational model of TAE outlined by Clifford *et al.*^2^.

### Computational model of TAE from GPs

A computational model of cortical adaptation was implemented to replicate the data gathered, based on the model proposed by Clifford *et al.*^2^. The underlying structure is a population of model neurons, each one exhibiting a preferred spatial orientation, and only responding to those components of the stimulus that are close to such orientation. The output of each neuron is described as a vector with a length proportional to the neuron response and parallel to the preferred orientation. The perceived direction is therefore given by the vector average of the outputs of the population.

The model acts on an internal space that is a representation of the physical plane on which the stimulus is displayed. In particular, horizontal and vertical directions are mapped as opposites in the internal space, so that 180° in model space corresponds to 90° in tilt. As a consequence, in the following we will always map an angle *θ* in the physical space in an angle *ϕ* = 2*θ* in the internal space. All the computations will therefore be carried out in the internal space, and then the result will be re-mapped in the physical space. We start by defining the response of a neuron oriented at an angle *θ*_*i*_ with respect to some Cartesian system of reference to a stimulus oriented at an angle *θ* as:


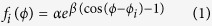


where *α* is the peak response (achieved when the preferred direction of the neuron is parallel to the stimulus) and *β* sets the width of the tuning curve. As reported by Clifford *et al.*^2^, the effect of a prior adapting stimulus is twofold. Without loss of generality, it will be assumed here that the adaptation is oriented at 0° with respect to the arbitrary system of reference. Then the first effect is to inhibit the response of the neurons tuned to nearby orientations, resulting in a modified peak response:


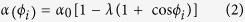


while the second is to broaden the bandwidth of the neurons tuned to oblique orientations, resulting in a modified width parameter:


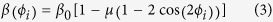


both *α*_0_ and *β*_0_ are defined as the unadapted values of the parameters, while *λ* and *μ* parameterize the magnitude of the two adaptation effects. It is argued in Clifford *et al.*^2^. That these two effects could be identified, respectively, with the centering and scaling mechanisms that take place in cortical neurons.

The combined effect of the two modifications is a departure of the perceived direction from the actual one. Such departure is defined as the difference between the initial angle *θ* and the result of the following computation. As previously stated, each adapted response function is interpreted as the length of the corresponding response vector:


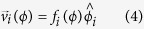


where the *versor* 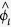 is the unit vector oriented at an angle *ϕ*_*i*_. Performing a vector average over all the preferred orientations


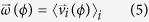


one ends up with a perceived angle


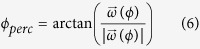


and the TAE in the physical space is finally defined as


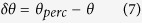


Such departure results to be repulsive as long as the difference between the adaptation and the test stimulus is small (i.e., when ≪90°), shifting towards attraction when the difference becomes larger. A best fitting procedure based on the implementation of a Metropolis-Hastings algorithm was then applied to the model in order to fit its outputs to the TAE data following adaptation to translational GPs. After ~5000 iterations, we obtained the following values for the parameters: *α*_0_ = 4.008, *β*_0_ = 4.052, *λ* = 0.035 and *μ* = 0.329. The total RMSE was 0.109. The resulting TAE curve, alongside the experimental data points, is shown in Fig. 2.

It should be noted that in the psychophysical data the adapting orientation is not constant as it ranges from 0° to 90°. This is only apparently different from the computational model explained so far. In fact, the arbitrariness of the choice of the reference system implies that the only actual dependence of the TAE can lie in the absolute difference between adaptation and test directions. The only reason why the convention of our model does not match that of the psychophysical data is that the former was chosen to match the original model of Clifford *et al.*^2^.

## **Experiment 2**: Interocular transfer of TAE from adaptation to GPs

The behavioral and computational results of Experiment 1 strongly suggest that TAE from GPs relies on early orientation selective mechanisms and their interactions, resulting in both repulsive and attractive effects. In Experiment 2, using the interocular transfer procedure (IOT), we psychophysically investigated whether orientation selective units responding to translational GPs are either monocular or binocular^17^,^49^. It is generally assumed that the strength of an aftereffect depends on how many neurons are adapted, thus the magnitude of IOT can give an indication of how many binocular or monocular neurons are involved during the adaptation process^50^. The extent of the transfer can be measured as a proportion of the magnitude of the aftereffect and ranges from 0%, which means no transfer, up to 100%, which implies a complete IOT. If there is 0% transfer, than the neurons stimulated are most likely monocular. The more transfer occurs, the more binocular neurons seem to be involved and higher cortical level of activation can be assumed^51^,^52^,^53^. Cells in the striate cortex show a wide range of binocularity, which means that they can be completely monocular, completely binocular or exhibit a range of binocularity^49^,^54^. Hypothetically, if an aftereffect transfers completely from one eye to the other, mostly binocular neurons must be involved^50^,^55^,^56^. If the IOT is not complete, for example by showing a smaller aftereffect in the non-adapted eye, it is assumed that monocular cells are also responding, thus modulating the amount of interocular transfer. An aftereffect that does not transfer from the adapted to the un-adapted eye imposes a process where only monocular cells are involved.

### Methods

#### Participants

One of the authors (JH) and an independent sample of seven new naïve participants took part voluntarily to the experiment, and all received compensation (except for the author). Seven participants were right eye dominant and one was left eye dominant. Ocular dominance was tested with a near-far alignment test^57^. In this test the observer holds a pencil in one hand in front of her/himself. Then, the observer has to align the tip with a point on a distant wall with both eyes open. The subject is then asked to alternatively close one eye. Only when the dominant eye is open and the other eye closed, the tip of the pencil will remain in good alignment with the point on the wall. This test was repeated with the pencil in the other hand. Methods were carried out in accordance with the Declaration of Helsinki (1964). This study was approved by the Ethics Committee of the University of Regensburg (reference number: 13-101-0029). Written informed consent was obtained from each participant prior to the enrolment in the study.

#### Stimuli and Procedure

**Figure 3:**
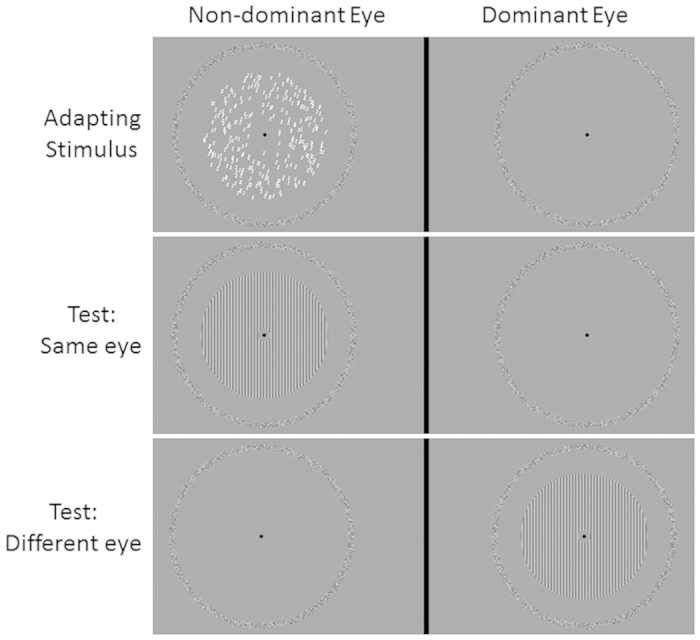
Representation of the procedure used in Experiment 2. The screen was divided by an opaque black card used as septum (illustrated here by a thick black continuous line). A custom mirror stereoscope (not represented) was used to fuse the images projected to the left and right eye. The circular noisy frames were used to aid fusion. The adapting stimuli were always presented to the non-dominant eye. The test grating could be presented either to the non-dominant eye (same eye condition) or to the dominant eye (different eye condition). Only an adapting GP is shown, but another group of observers were adapted to a tilted grating.

Apparatus and stimuli were the same as in Experiment 1. In Experiment 2 we implemented an IOT procedure using a custom mirror stereoscope. The computer screen was halved using an opaque black card as septum, this ensured that each eye viewed its respective half of the screen (Fig. 3). On each half of the screen a circular noise frame was presented to facilitate the fusion of the images from the two eyes. The inner radius of the circular frame was 6.8 deg, whereas the outer radius was 7.3 deg. The center-to-center distance of the two circular frames was 25.5 deg. Adapting and test stimuli were the same as used in Experiment 1. The procedure was also the same as reported for Experiment 1 with the exception that the adapting stimuli were always presented to the non-dominant eye, whereas the test pattern was either presented to the non-dominant (same eye condition) or to the dominant eye (different eye condition)^58^. Moreover, we only adapted to a translational GP oriented 15° clockwise from vertical, since this adapting angle produced the strongest direct TAE in Experiment 1.

In a second part of the experiment we also assess the IOT of the TAE following adaptation to a tilted grating. One of the authors (JH) and an independent sample of five naïve participants were adapted to a grating tilted 15° clockwise from vertical. The adapting grating had the same spatial characteristics of the test grating. The procedure was the same as described for GPs except that the adaptation-test blank interval was set at 300 ms instead of 16 ms. This avoided any feed-forward masking between adapter and test stimulus.

### Results

**Figure 4:**
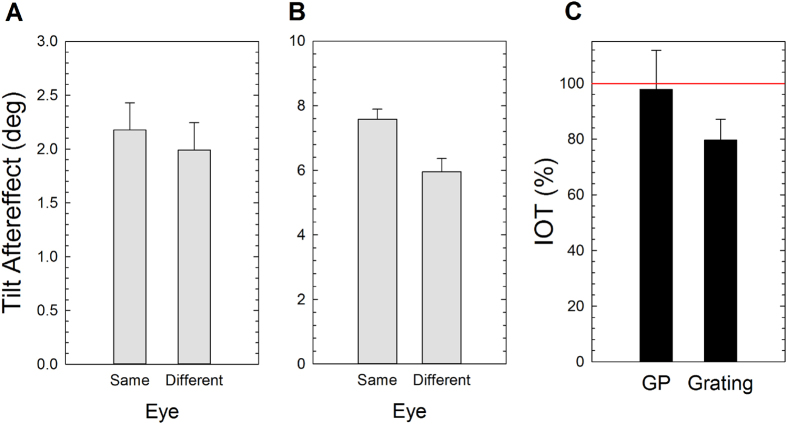
Results of Experiment 2. (**A**) TAE (in deg) estimated following adaptation to translational GPs oriented at 15° clockwise from vertical. The TAE is plotted for the test eye (i.e., same vs. different from the adapted eye). (**B**) TAE estimated following adaptation to gratings tilted 15° clockwise from vertical (note different ordinate scales for the GP and grating adapters). (**C**) The IOT of TAE from both GP and grating. Error bars ± SEM.

Figure 4 (panels A,B) shows the results of Experiment 2. A mixed ANOVA including the between-subjects factor Stimulus Type (GP vs. grating) and the within-subjects factor the Test Eye (same vs. different eye) revealed a significant effect of the Stimulus Type (*F*_1,12_ = 178.05, *p* = 0.0001, *partial-η*^*2*^ = 0.94), a significant effect of the Test Eye (*F*_1,12_ = 8.71, *p* = 0.012, *partial-η*^*2*^ = 0.42) and a significant interaction between Stimulus Type and Test Eye (*F*_1,12_ = 5.51, *p* = 0.037, *partial-η*^*2*^ = 0.32). Bonferroni corrected pairwise comparisons revealed a significant difference between same and different eye only for gratings (*p* = 0.004). Subsequently we calculated the IOT index for each observer as follows:


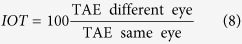


Figure 4C depicts the average IOT calculated for both stimuli. Although a Mann-Whitney test for two independent samples did not reveal a significant difference between the IOT estimated from adaptation to translational GPs and gratings (*Z* = −0.90, *p* = 0.41), a one-sample Wilcoxon Signed Rank test indicated that the IOT for translational GPs did not differ from 100% (*Z* = −0.21, *p* = 0.84) (IOT: 97.8%, SE: 14.08%). On the other hand, the IOT of the TAE from gratings was partial (IOT: 79.7%, SE: 7.43%) and significantly below 100% (*Z* = −2.0965, *p* = 0.031).

### Discussion

The results of Experiment 2 showed an almost complete IOT (97.8%) of the TAE following adaptation to translational GPs, and a partial IOT (79.7%) of the TAE from gratings. These results suggest that the TAE from GPs may rely on orientation selective units that are mostly binocularly driven^16^,^17^. On the other hand, the incomplete IOT of the TAE from gratings suggests adaptation of both monocular and binocular mechanisms^50^,^55^,^56^,^59^.

## **Experiment 3**: The role of attention in TAE from GPs

There is psychophysical evidence that the TAE is modulated by attention^38^,^60^,^61^. Bahrami *et al.*^60^ showed that, using a high load distracting task during orientation-specific adaptation to an invisible grating abolished almost completely the threshold elevation after-effect (TEAE); a variant of the TAE in which adaptation to a tilted grating increases the contrast detection threshold of test gratings of the same tilt and decreases the contrast threshold for the orthogonal tilt^60^,^62^,^63^,^64^. Moreover, Spivey and Sprin^38^ and Jung and Chong^61^ found that attention increases the amount of TAE when the adapters were visible. In Experiment 3 we investigated whether TAE from GP is also modulated by attention and whether distraction during the adapting phase differently affects TAEs from GPs and gratings since they are likely to involve different neural substrates.

### Stimuli and Procedure

The same participants of Experiment 2 also took part to Experiment 3. Methods were carried out in accordance with the Declaration of Helsinki (1964). This study was approved by the Ethics Committee of the University of Regensburg (reference number: 13-101-0029). Written informed consent was obtained from each participant prior to the enrolment in the study.

Stimuli and procedure of Experiment 3 were the same as Experiment 2, except for the addition of an attention-control task presented to the dominant eye. The attention-control task was the same to that used by Pavan and Greenlee^65^. It consisted of a rapid serial visual presentation (RSVP) of letters and digits. We used all the letters of the alphabet and digits from 1 to 4 and from 6 to 9. Letters and digits were presented in Arial font and subtended 0.6 deg. Letters and digits appeared for 250 ms interleaved with 150 ms with a blank (presentation rate: 2.5 Hz). After the images from the two eyes were fused, observers perceived the letter/digit stream at the center of the adapting stimulus. Observers were instructed to consider digits as the critical stimuli and letters as distracters and to respond as fast as possible whether a presented digit was above or below “5”. The keyboard keys were “X” to indicate digits >5 and “Y” to indicate digits <5 (keys refer to a standard German keyboard). The attention-control task was performed with the left hand and it was only performed during the adaptation period. Letters and digits were randomly chosen but with the constraint that two identical letters or digits could not be presented consecutively. To ensure high attentional load^60^, we used a rate of 1/3 of digit presentation (i.e., digits among letters presented)^58^,^65^. After the adapting phase the test grating was presented. Observers judged whether the test grating was tilted clockwise or counterclockwise from vertical (method of single stimuli, MSS). The keyboard keys were the same as those used in Experiment 1 and 2, and observers used the right hand to judge the orientation of the test pattern.

### Results

**Figure 5:**
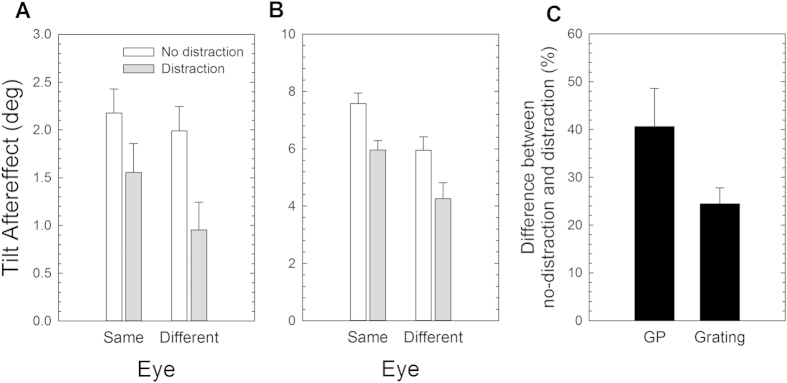
Results of Experiment 3. Tilt aftereffect (in deg) following adaptation to translational GPs (**A**) and gratings (**B**) oriented 15° clockwise from vertical as a function of the test eye. White bars represent data of Experiment 2. Note difference in ordinate scales in panels (**A–C**) Distraction effect on the TAE following adaptation to GPs and gratings. The percentage decrement of TAE induced by distraction was calculated for each observer and separately for GPs and gratings. Error bars ± SEM.

Figure 5 shows the results of Experiment 3. Panels A and B show the results obtained from GPs and gratings. Since the same observers took part to both Experiments 2 and 3 in the statistical analysis we also included data of Experiment 2 (i.e., no distraction condition). However, since a mixed ANOVA revealed a significant effect of the Stimulus Type (*F*_1,12_ = 223.4, *p* = 0.0001, *partial-η*^*2*^ = 0.95) we analysed data separately for GPs and gratings.

For GPs, a repeated-measures ANOVA revealed a significant effect of Attention (*F*_1,7_ = 8.48, *p* = 0.023, *partial-η*^*2*^ = 0.55), but no significant effect of the Test Eye (*F*_1,7_ = 1.51, *p* = 0.26, *partial-η*^*2*^ = 0.18), nor a significant interaction between Attention and Test Eye (*F*_1,7_ = 1.01, *p* = 0.35, *partial-η*^*2*^ = 0.13). Although the interaction Attention x Test Eye was not significant we also performed post-hoc Bonferroni corrected t-tests and found only a significant difference between the non-distracted and distracted conditions when the test pattern was presented to the unadapted eye (*p* = 0.018).

For gratings, a repeated-measures ANOVA revealed a significant effect of Attention (*F*_1,5_ = 52.7, *p* = 0.001, *partial-η*^*2*^ = 0.91), a significant effect of the Test Eye (*F*_1,5_ = 7.40, *p* = 0.042, *partial-η*^*2*^ = 0.60), but a non-significant interaction between Attention and Test Eye (*F*_1,5_ = 0.026, *p* = 0.88, *partial-η*^*2*^ = 0.005). Also in this case, although the interaction Attention x Test Eye was not significant we performed a series of post-hoc Bonferroni corrected t-tests. The t-tests pointed out that for the non-distracted condition there was a significant difference between the same and different eye conditions (*p* = 0.048). The same effect was evident when attention was diverted during adaptation (*p* = 0.05). In addition, the t-tests also revealed a significant difference between the non-distracted and distracted conditions when the test pattern was presented to the adapted and unadapted eye (*p* = 0.004 and *p* = 0.002, respectively).

We conducted a subsequent analysis aimed to assess whether distraction affected differently TAEs for GPs and gratings. In particular, we pooled data across the test-eye factor (despite a significant effect of this factor when adapting to gratings). Then for each observer we calculated the percentage decrement of the TAE between the no-distraction and the distraction condition, separately for GPs and gratings. The results indicate the extent to which the RSVP fixation task affected the TAE following GP and grating adaptation (Fig. 5C). A Kolmogorov-Smirnov test did not point out any significant difference between the effect of distraction estimated for GPs and gratings (*Z* = 1.16, *p* = 0.093). The non-significance could imply that we are lacking statistical power for this comparison.

### Discussion

The results of Experiment 3 show that diverting attention away from the adapter with a demanding RSVP task as a distractor, reduces the magnitude of the TAE for both GPs and gratings. These results are in agreement with previous findings that selective visual attention influences adaptation to visual orientation information^38^,^61^. Although the distraction-induced reduction in the TAE did not obtain statistical significance owing to the low power of this comparison, distraction reduced the TAE for GP adaptation by 40.6%, and by 24.5% after grating adaptation. These results suggest that attention may affect differently orientation adaptation from translational GPs and gratings. However, further studies with larger sample sizes will be required before any final conclusions can be drawn here.

## **Experiment 4**: The role of attention in GP coherence threshold

From Experiment 3 it is not clear whether the distraction-induced reduction of the TAE from GPs depends on the fact that distraction affects the spatial summation of local orientation signals from the presented dipoles or whether it affects the adaptation process itself. To this purpose we performed another experiment in which we assessed the effect of attention on the coherence threshold of GPs. The rationale behind this experiment was that if distraction affects the spatial summation, then diverting attention during adaptation away from coherent stationary translational GPs, should increase the coherence threshold of a subsequently viewed test GP more than in the non-distracted condition. On the other hand, no effect of distraction on the coherence threshold of the test GP (i.e., no difference between the attention distracted and non-distracted conditions) would indicate that distraction is likely to modulate the adaptation process itself. Experiment 4 is based on previous psychophysical evidence that adaptation to GPs affects the signal strength (in terms of coherently oriented local dipoles) according to which test GPs would be classified by our participants^24^,^66^.

### Participants

One of the authors (AP) and five naïve participants took part voluntarily to the experiment with no compensation. All participants had normal or corrected to normal visual acuity. Viewing was binocular. Methods were carried out in accordance with the Declaration of Helsinki (1964). The experiment was approved by the ethical committee of the University of Lincoln (reference number: PSY1415124). All participants gave written informed consent prior to their inclusion in the experiment.

### Apparatus

Stimuli were displayed on a 20-inch HP p1230 monitor with a refresh rate of 85 Hz. Stimuli were generated with Matlab PsychToolbox^39^,^40^. The screen resolution was 1280 × 1024 pixels. Each pixel subtended 1.6 arcmin. The minimum and maximum luminance of the screen were 0.08 and 74.6 cd/m^2^ respectively, and the mean luminance was 37.5 cd/m^2^. A gamma-corrected lookup table (LUT) was used so that luminance was a linear function of the digital representation of the image. Observers sat in a dark room at a distance of 57 cm from the screen. The participant’s head was stabilized by asking her/him to rest her/his chin on a chinrest.

### Stimuli and Procedure

Adapting stimuli were vertically oriented translational GPs (100% coherence) with the same spatial characteristics of the GPs described in previous Experiments. The test pattern was a GP vertically divided in two halves. One half of the test pattern (composed by 150 dipoles) contained randomly oriented dipoles (0% coherence), whereas the other half of the test pattern contained vertically oriented dipoles whose coherence was manipulated using a simple up/down staircase^43^. The relative positions of the stimulus halves were varied randomly from trial to trial. After adaptation to a coherent GP, observers judged which half of the test pattern was more coherent (two-alternative forced-choice task; 2AFC). A simple up-down staircase varied the coherence level (in terms of vertically oriented dipoles) of the test GP until the observers were no longer able to discriminate which half of the test was more coherent (i.e., point of subjective equality; PSE). In particular, the staircase always started from the condition in which one half of the test GP had 0% coherence and the other half had 100% coherence and varied only the coherence of the latter half.

Experiment 4 consisted of three conditions: (i) a baseline condition in which there was no adaptation and we estimated the PSE individually for each observer, (ii) a condition in which observers were adapted to a vertical translational GP, and (iii) a condition in which observers during adaptation to a vertical translational GP also performed a secondary task.

In the baseline condition, the test stimulus was presented for 500 ms and observers had to judge which half of the test stimulus was more coherent. The staircase terminated either after 200 trials or 20 reversals. The coherence threshold was calculated by averaging the coherence level corresponding to the last twelve reversals.

In the adaptation only condition, the procedure was the same as in the baseline condition with except that observers were adapted to a vertical translational GP. We use a top-up adaptation procedure in which the first adapting stimulus was presented for 30 s and the other adapting stimuli for 10 s. In the adaptation plus distraction condition, the procedure was the same as in the adaptation only condition with the exception that a secondary distracting task was presented at fixation for the entire duration of the adaptation (i.e., 30 or 10 s). The distracting task was the same as that used in Experiment 3.

### Results

**Figure 6:**
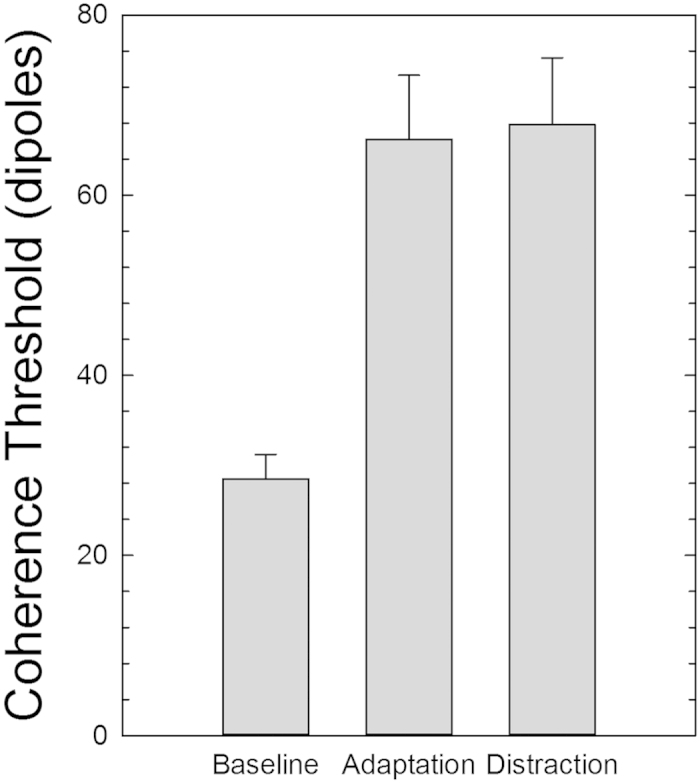
Coherence threshold expressed as the number of vertically oriented dipoles necessary to discriminate which part of the test pattern was more coherent. Coherence thresholds are reported for each experimental condition. Error bars ± SEM.

Figure 6 shows the results of Experiment 4. A one-way repeated measures ANOVA reported a significant effect of the adapting condition (*F*_2,10_ = 28.25, *p* = 0.0001, *partial-η*^*2*^ = 0.85). Simple contrasts revealed a significant difference between the baseline condition and both adaptation conditions (*F*_1,5_ = 28.31, *p* = 0.003, *partial-η*^*2*^ = 0.85 and *F*_1,5_ = 33.09, *p* = 0.002, *partial-η*^*2*^ = 0.87 for the adaptation only and adaptation plus distraction conditions, respectively). However, a repeated contrast did not reveal a significant difference between the two adaptation conditions (*F*_1,5_ = 0.31, *p* = 0.60, *partial-η*^*2*^ = 0.06). These results suggest that distraction during adaptation does not affect the spatial summation of local dipoles. Therefore, in the case of the TAE, distraction seems to modulate the orientation adaptation process, but does not affect the effect of adaptation on GP coherence thresholds. Adaptation does, however, have a robust effect on the coherence thresholds for both distracted and non-distracted conditions^24^,^66^.

In order to assess the significance of the above comparison, it is necessary to evaluate the performance floor of the measured effect. The same simple up/down staircase was applied to a simulated observer giving random responses, and the coherence threshold was extracted. As expected, it resulted to be slightly below 100%. Averaging over 1000 repetitions of such procedure, we obtained a mean random coherence threshold of 125.4 (SD: 23.9), which is significantly different from the coherence threshold estimated in the baseline condition (*t*_992_ = −9.89, *p* = 0.0001), in the non-distracted condition (*t*_992_ = −6.03, *p* = 0.0001), and in the distracted condition (*t*_992_ = −5.87, *p* = 0.0001).

## General Discussion

In this study we investigated whether prolonged exposure to static oriented translational Glass patterns (GPs) can adapt orientation selective mechanisms. The effect of adaptation to translational GPs was probed measuring the tilt aftereffect (TAE). The results of Experiment 1 showed the typical angular function of the TAE^2^,^7^,^19^,^45^,^67^. In particular, we found significant direct TAE effects when adapting at 15° clockwise from vertical (direct TAE: 1.73° and 1.3°). The magnitude of the peak direct TAE reported in this study is reduced in amplitude with respect to direct TAEs reported in previous studies using oriented gratings or lines. For example, Mitchell and Muir^7^ found a direct effect ranging from 4° to 6° and an indirect TAE around 2°–3°. In our Experiment 2 we also reported a much stronger direct TAE oriented grating (i.e., 7.57°, SE: 0.37°). However, the magnitude of the peak for direct TAE after adaptation to GPs is, on average, similar to the direct TAEs estimated in experiments in which observers were adapted to oriented subjective contours^16^,^17^, texture edges defined by orientation contrast^16^, and bilateral symmetrical patterns^18^. For example, Paradiso *et al.*^17^ reported a peak direct TAE ranging between 2° and 3° when observers were adapted to “pure” subjective contours. In our case the direct TAE ranged between 0.86° and 3° when adapting to translational GPs. One possible explanation of the difference in magnitude between TAE induce by GPs and gratings is that though local dipoles generate oriented responses in the striate cortex, the orientation bandwidth of the response is greater for GPs than gratings^31^,^32^, and this could potentially affect gain control and inhibition mechanisms at all stages of processing. Another reason could be that dipoles have lower contrast energy than gratings in any given spatial frequency band^68^, thus producing weaker responses in orientation selective units^31^.

In our psychophysical data we also reported a very weak (and not statistically different from zero) indirect TAE (magnitude: −0.19°). This could depend on the fact that the direct TAE after adaptation to GPs was reduced in amplitude, the indirect TAE would be, in turn, even smaller. The indirect TAE is a relevant component of the angular function describing the TAE; weak indirect TAEs have been reported in most of the studies involving adaptation to oriented stimuli^2^,^7^,^9^,^16^,^19^,^45^,^67^,^69^. The nature of the indirect TAE is not yet well understood, but there is computational evidence that the indirect TAE can be obtained by the same inhibitory interactions between orientation selective neurons. To this purpose, our computational model was able to describe both direct and indirect effects (see Fig. 2), suggesting that both effects may depend on units tuned for different orientations and their interactions. It is should be noted that the qualitative nature of the model does not allow us to formulate predictions regarding the form and magnitude of the angular function, whose parameters were in fact estimated by means of a best fitting routine. Quantitative predictions could be achieved through a computational model involving a description in the frequency domain of the system response to the selected visual stimuli^70^,^71^, as well as a mathematical mechanism mimicking adaptation at the level of local orientation detectors. However, such analysis lies beyond the scope of the present study, and it is postponed to a forthcoming study.

In Experiment 2 we measured the IOT of the TAE from translational GPs, and compared it with the IOT of the TAE estimated after adaptation to gratings. The results showed that the direct TAE following GP adaptation transfers almost completely from the adapted eye to the unadapted eye (IOT: 97.8%), whereas the IOT for the direct TAE after grating adaptation was partial (79.7%), being in line with the IOT reported by other studies, and ranging between 50–80%^52^,^55^,^56^,^72^,^73^. Although the IOT values estimated after adaptation to GPs and gratings did not differ significantly from each other, the IOT estimated after GPs adaptation did not differ from 100% (i.e., complete IOT), whereas the IOT estimated from gratings adaptation was significantly below 100%. These results suggest that TAE from static translational GPs is likely to be mediated by neurons that are mostly binocularly driven. On the other hand, the IOT from gratings also show a quite high transfer to the unadapted eye, though it was not complete. This suggests the involvement of both monocular and binocular neurons. These findings are in agreement with previous IOT findings of Vreven and Berge^74^. These authors found higher IOT following adaptation to dynamic GPs compared to that found after adaptation to stationary GPs. However, they did not measure the TAE but adapted to static or dynamic GPs and examined the modulation of the coherence threshold on test GPs. Nevertheless, the authors concluded that binocularly driven detectors contribute to the perception of structure in both static and dynamic GPs. Clifford and Weston^24^ also measured the IOT of the aftereffect of adaptation to concentric GPs and found an IOT of 53%, suggesting the involvement of both monocular and binocular units. In contrast, the IOT of the TAE from translational GPs reported in the present study is likely to depend on mostly binocularly driven units^74^. Based on the computational model outlined by Clifford^2^, the aftereffect reported by Clifford and Weston^24^ is likely to rely on a centering mechanism only, whereas the TAE from translational GPs may involve centering-and-scaling.

From our psychophysical data it is not possible to infer in which areas translational GPs are encoded and where the dichoptic adaptation takes place. To this purpose there is recent human brain imaging evidence of monoptic and dichoptic adaptation (i.e., IOT) to gratings in the striate cortex and extrastriate areas like V2 and V3^59^,^75^. In addition, there is physiological evidence in macaque monkeys and human brain imaging evidence that orientation selective neurons exist in a wide range of extrastriate areas. For example, Fang *et al.*^14^ found that after long-term adaptation to an oriented grating, fMRI response in visual areas V1, V2, V3/VP, V3A, and V4 to a test stimulus was proportional to the angular difference between the adapting and test stimuli. Based on these results, it could be that TAE from translational GPs may rely on multiple cortical sites in which orientation adaptation takes place (i.e., from V1 up to V4). Furthermore, fMRI studies with monkeys and humans have shown that GP detection involves global integration of local orientation and shows activation of neurons in area V4^76^,^77^. For example, Tse *et al.*^76^ found that concentric, radial, translational, and random GPs produced substantial activity in V1 and V2, and in some cases also in more anterior areas including V4. Further analysis reported that concentric patterns elicited greater activation than other patterns, and this difference was most marked in anterior extrastriate cortical areas including V4, suggesting that these areas contain neurons selective to different global forms. Ostwald *et al.*^35^ found fMRI selectivity for translational GPs across a range of visual areas including V1, V2, VP, V3/V3A, V4 and LOC. Some of these findings are in agreement with previous multistage models of GP perception^26^,^34^ in which V1/V2 neurons perform the local analysis of dipoles’ orientation^26^,^31^,^32^ while the spatial integration of signals is possibly achieved in more anterior areas, possibly including the V3 complex and area V4^26^,^34^,^77^, whereas other suggest that global form can be extracted as early as primary visual cortex^36^. An alternative explanation of why adaptation to translational GPs elicits the TAE is that by integrating the dot pairs to a global form in higher visual areas this information could be send back as feedback to lower visual processing areas selective for orientation (e.g., V1). This view appears to get support from the findings of Chen *et al.*^78^ who found that global contours are processed in a parallel manner in both V1 and V4. Furthermore, they found a slightly earlier onset (40 ms) of V4 activation compared to V1 activation. Their results suggest an integration process that uses feed forward and feedback signals to enhance global structure and suppress background noise. Therefore, it seems plausible that feedback from higher areas is active in modulating low-level encoding of orientation.

In Experiment 3 we tested whether diverting attention away from adaptation to translational GPs affected the resulting TAE. In agreement with previous studies on the attentional effects on TAE from gratings^38^,^60^,^61^ we found a distraction-related decrement of the TAE following adaptation to GPs and gratings. The attentional reduction was found either when both adapting and test patterns were presented to the same eye or when they were presented to different eyes. These results were expected since there is fMRI evidence that attention influences low-level processing as well as high-level processing^79^,^80^. However, we found that distraction decreased the direct TAE after GP adaptation by 40.6%, compared to a reduction of 24.5% in the case of the direct TAE after grating adaptation. Although this difference was not statistically significant, it may be possible that attention differently modulates orientation adaptation from GPs and gratings. If this is the case, it might depend on the processing levels of translational GPs, as the attentional effects become more pronounced in higher-order visual areas^81^,^82^,^83^.

Another important result comes from Experiment 4 in which we tested the effect of distraction during GP adaptation on the coherence threshold of test GPs. The results showed that, while adaptation had a striking effect on coherence thresholds for detecting the orientation of GPs, performing a distracting task during adaptation had no effect on the coherence threshold of the test pattern; the average threshold, in terms of coherently oriented dipoles, necessary to discriminate which part of the test GP was more coherent was identical in the non-distracted and distracted conditions. These results suggest that the extraction of the global form/orientation in translational GPs is pre-attentive, and that attention modulates the adaptation process rather than interfering with orientation pooling mechanisms.

## Additional Information

**How to cite this article**: Pavan, A. *et al.* Tilt aftereffect following adaptation to translational Glass patterns. *Sci. Rep.* **6**, 23567; doi: 10.1038/srep23567 (2016).
